# Decreased Levels of Soluble CD44 in a High-Risk Population following a Smoking Cessation Program

**DOI:** 10.3390/ijerph182413174

**Published:** 2021-12-14

**Authors:** Drew H. Smith, Shahm Raslan, Isildinha M. Reis, Abdurrahman Al-Awady, Isabella Buitron, Melanie Perez, Huaping Liu, Jerri Halgowich, Claudia Gordon, Monica Webb Hooper, Noël C. Barengo, Elizabeth J. Franzmann

**Affiliations:** 1 Department of Otolaryngology—Head and Neck Surgery, University of Miami Leonard Miller School of Medicine, Miami, FL 33136, USA; drew.h.smith@ttuhsc.edu (D.H.S.); srasl001@med.fiu.edu (S.R.); abdurrahman.al-awady@med.miami.edu (A.A.-A.); isb279@nyu.edu (I.B.); melyp2001@hotmail.com (M.P.); hliu2@med.miami.edu (H.L.); jhalgowich@med.miami.edu (J.H.); 2 Herbert Wertheim College of Medicine, Florida International University, Miami, FL 33199, USA; 3 Sylvester Comprehensive Cancer Center, University of Miami Leonard Miller School of Medicine, Miami, FL 33136, USA; ireis@med.miami.edu (I.M.R.); cgordon2@med.miami.edu (C.G.); 4 Department of Public Health Sciences, University of Miami Leonard Miller School of Medicine, Miami, FL 33136, USA; 5 New York University, New York, NY 10003, USA; 6 Case Comprehensive Cancer Center, Case Western Reserve University, Cleveland, OH 44016, USA; monica.hooper@nih.gov; 7 Department of Translational Medicine, Herbert Wertheim College of Medicine, Florida International University, Miami, FL 33199, USA; nbarengo@fiu.edu; 8 Department of Health Policy and Management, Robert Stempel College of Public Health and Social Work, Florida International University, Miami, FL 33199, USA

**Keywords:** solCD44, cotinine, total protein, head and neck cancer, tobacco, smoking intervention

## Abstract

Tobacco is a risk factor of head and neck cancer (HNC) and smoking cessation alone may reduce HNC risk by 70%. Soluble CD44 (solCD44), a cell surface receptor linked to cell proliferation and migration, and total protein (TP) levels can detect early HNC. This study aims to determine whether salivary solCD44 and TP levels in oral rinses change following a smoking cessation program. 150 smokers provided oral rinse samples at baseline and at a 12-month follow-up after participation in a smoking cessation program. Assays to measure levels of solCD44, TP, and cotinine, a metabolite used as a biomarker of tobacco exposure, were completed. A paired-samples t-test was used to determine whether there was a statistically significant (*p* < 0.05) mean difference in biomarker levels before and after the program. Baseline and at 12-month follow-up data were available for 88 subjects, 21 of whom quit smoking entirely. Mean levels of solCD44 significantly decreased by 0.412 ng/mL from baseline to the 12-month follow-up, *p* = 0.010. There was no significant difference in mean TP levels, *p* = 0.975. Mean cotinine levels decreased significantly by 74.7 ng/mL, *p* = 0.035. This is the first work demonstrating an association between smoking cessation and decreased solCD44 levels in oral rinses. Decreased expression of the tumorigenic CD44 may be one mechanism by which smoking cessation lowers cancer risk.

## 1. Introduction

Head and neck cancers (HNC) are among the most common malignancies worldwide, surpassing 900,000 cases and 400,000 deaths per year [1]. Specifically, these cancers affect the squamous cells that line the mucosal surfaces of the head and neck; regions such as the oral cavity, nasal cavity, oropharynx, and larynx are most susceptible to carcinoma [2]. Tobacco and alcohol consumption have long been recognized as significant risk factors [1,3]. Although individuals who use both tobacco and alcohol are at greater risk of developing these cancers than those who use either tobacco or alcohol alone, the single largest risk factor for head and neck cancer is tobacco, which has been linked to over 50% of all cases in the United States [4]. Individuals classified as heavy cigarette smokers experience up to a 25-fold increased risk of HNC compared with nonsmokers, and a dose-response relationship is evident [1]. Studies have shown that smoking cessation alone could reduce the risk of HNC by 70% [5]. Furthermore, the best predictor for progress to cancer among premalignant lesions is the severity of dysplasia, a histologic diagnosis [6]. Studies have even shown that smoking cessation can reverse dysplasia [7,8,9].

Various biomarkers for head and neck squamous cell carcinoma exist, including CD44. CD44 is a transmembrane glycoprotein expressed in a variety of cells such as embryonic stem cells, connective tissue, and bone marrow, and has shown to be involved in cell proliferation, migration, and tumor initiation [10,11,12,13,14]. Since CD44 expression is upregulated in certain populations of cancer cells. It can be used as a molecular marker, especially for cancer stem cells [11,12,15]. With its central role in cancer stem cells, CD44 may also be utilized as a prognostic marker of tumor regeneration following therapy [14]. Our lab has previously published the viability of using salivary soluble CD44 (solCD44) and total protein levels for the early detection of oral and oropharyngeal cancer [16,17]. Furthermore, we have demonstrated that higher levels of solCD44 and total protein are inversely correlated with progression free survival and overall survival among oral and oropharyngeal cancer patients [18].

Many variants of CD44 exist [19,20]. CD44 isoforms CD44v6 and CD44v5 were shown to decrease rapidly in serum after the cessation of smoking, but the combination of all the variants did not change significantly [21]. SolCD44 levels in serum are largely contaminated by variant isoforms derived from normal epithelial compartments, thus serum may not be the most suitable body fluid to study. Furthermore, serum collection is much more invasive than simply collecting saliva or an oral rinse. In addition to the dearth of salivary studies, previous smoking cessation and CD44 studies have also featured small sample sizes, lacked measurement of cotinine levels after one year, and were deficient in univariate and multivariate analysis.

The objective of this project was to determine whether there was a change in salivary solCD44 and total protein levels in oral rinses following a smoking cessation program.

## 2. Materials and Methods

### 2.1. Study Design and Participants

This one group experimental study enrolled 150 active smokers from 2011 to 2014 through community organizations and a community-based research study in north Miami, FL. These participants were considered high-risk for development of head and neck cancer based on smoking status. Participation was voluntary and enrollment featured convenience sampling. Inclusion criteria were a status of currently smoking cigarettes daily, willingness to enroll in a smoking cessation program and provide oral rinse samples at a 12-month follow-up visit, and age greater than 21, although preference was given to smokers over age 40. Those with a personal history of previous cancer or smokers who quit more than one week prior to entry in the study were excluded.

### 2.2. Assessment of Study Variables

All participants completed a questionnaire at baseline and again at the 12-month follow-up, which included questions regarding demographics (age, gender, education level), subjective oral health, number of teeth removed, number of alcoholic drinks per day, dietary habits (green salad and vegetable intake), and smoking history. Smoking history was further detailed by number of cigarettes smoked per day, age at initial use of tobacco, number of years smoking, experimentation with other types of tobacco including cigars and chewing tobacco, and exposure to secondhand smoke at home and in the workplace. All variables were self-reported through the questionnaires.

### 2.3. Assays

Subjects provided oral rinse samples at baseline and at a 12-month follow-up after participation in a smoking cessation program. Levels of solCD44 were measured using a sandwich ELISA assay (eBioscience, San Diego, CA, USA), with previously published modifications [12,16,17,22]. Total protein levels were measured with the DC protein assay (Bio Rad Laboratories, Hercules, CA, USA) according to the manufacturer’s protocol using saliva samples prepared as previously published [12,16,17,22]. Levels of cotinine, a metabolite used as a control biomarker for tobacco exposure, were measured by an ELISA assay (Salimetrics, State College, PA, USA). All assays were completed in the lab of Dr. Elizabeth Franzmann at the University of Miami Department of Otolaryngology. Lab technicians were blinded to smoking history and cessation program status of participants.

### 2.4. Smoking Cessation Program

The smoking cessation program was developed under the direction of the University of Miami Department of Psychology and the Sylvester Comprehensive Cancer Center. Smokers were provided resources including eight sessions of group-based cognitive behavioral treatment, health education, eight weeks of nicotine replacement therapy, and then were followed up to one year. Other options offered included Florida Smokers’ Quit line and primary care visits. Monthly contact was maintained with participants.

### 2.5. Statistical Analysis

The paired-samples t-test was used to determine whether there was a difference in biomarker levels before and after the smoking cessation program. Two-sample t-tests were used to determine whether the changes from baseline in biomarker (solCD44, TP, cotinine) were different in two groups defined by a selected variable. Analysis of variance (ANOVA) was used for comparison of mean marker change from baseline among three or more groups. A multivariable model for the outcome marker change from baseline was developed testing selected variables using stepwise selection procedure with *p* < 0.30 to enter and *p* < 0.10 to stay in model. The McNemar’s Chi-Square Test was performed to assess for marginal homogeneity between dichotomous variables at baseline and at 12 months. Bowker’s Test was used to assess for symmetry of frequencies at baseline and 12 months for the self-reported number of cigarettes smoked per day as an ordinal variable. Statistical analyses were performed using SAS version 9.4 (SAS Institute, Inc., Cary, NC, USA) with *p* ≤ 0.05 considered statistically significant.

### 2.6. Ethical Considerations

This study was approved by the University of Miami Institutional Review Board through its Human Subject Research Office. Each participant voluntarily signed an approved informed consent form and understood the he or she could withdraw his or her participation at any point in the study.

## 3. Results

Eighty-eight participants who remained in the study for the full twelve months and provided scheduled oral rinses were included for the statistical analysis. The majority of participants were under age 60 (87.5%), male (53.4%), had an education level less than or equal to grade 12 or GED (70.5%). 88.6% of participants reported unemployment vs. an occupation with some income (11.4%). 92.0% of participants reported less than $25,000 of annual income, with 6.8% uncertain or refusing to report income levels. Table 1 describes the changes in risk factors including oral health, drinking habits, nutrition, and tobacco use of the 88 participants at baseline and 12 months. At baseline, the participants were smoking an average of 11.2 cigarettes per day, with a range from 2 to 40. Twelve months into the smoking cessation program, the average number of cigarettes smoked per day decreased significantly to 3.8 (*p* < 0.001). Twenty-one of the eighty-eight (23.9%) participants quit smoking completely.

We next investigated whether there were changes in covariates other than tobacco use that could potentially confound results. There was no statistically significant difference between the proportions of participants with good oral health at baseline versus at the 12-month follow-up assessment (48.9% vs. 53.4%, *p* = 0.206, McNemar’s test). No statistically significant difference was found between participants with no/1–5 teeth removed at baseline versus at 12 months (48.9% vs. 45.5%, *p* = 0.180). There was no statistically significant difference between the proportions of participants with no/mild alcohol consumption at baseline versus at 12 months according to previously published criteria [23] for alcohol consumption (18.2% vs. 15.9%, *p* = 0.625). We recently found an inverse association between CD44 levels and green salad/vegetable intake [23]. Here, no statistically significant difference was found between baseline and 12-month values for the proportions of participants consuming less than one or zero salads per week (39.8% vs. 38.6%, *p* = 0.705), consumption of other vegetables less than once per week (5.7% vs. 6.8%, *p* = 1.000), consuming green salad or other vegetables less than once per week or never (2.3% vs. 4.5%, *p* = 0.500), and consuming green salad and other vegetables less once per week or never (66.7% vs. 33.3%, *p* = 0.508).

Marker levels were assessed at baseline and at the 12-month assessment. Data are mean ± standard deviation as displayed in Table 2. Levels of solCD44 were significantly lower (1.405 ng/mL ± 0.963 at 12 months compared to levels at baseline 1.816 ng/mL ± 1.280, *p* = 0.0103). There was no significant difference between mean TP level at the 12-month follow-up (0.497 mg/mL ± 0.348) versus baseline (0.496 mg/mL ± 0.328), *p* = 0.975. Levels of the cotinine control, available from 55 of the 88 subjects, decreased (196.7 ng/mL ± 212.7 at 12 months compared to 271.5 ng/mL ± 322.0 at baseline, *p* = 0.035).

We performed a univariable analysis including multiple covariates such as age and gender as well as oral health, tobacco use, alcohol use and nutrition at baseline and 12 months to determine factors that may influence marker level changes from the baseline to the 12-month assessment. Selected results for CD44 are shown in Table 3, while full results for CD44, cotinine and TP can be found in Supplementary Tables S1–S3, respectively.

As indicated by the 95% confidence intervals in Table 3 for change from baseline, significant decrease at 12 months compared to baseline was obtained for subjects with poor/fair oral health, for those who reported smoking >10 cigarettes per day at baseline and for those with cigarettes smoked per day at 12 months >0 to 5 or >0. When compared the subject subsets, a significantly greater difference in mean decrease in solCD44 levels was observed in those with poor/fair oral health compared with good oral health (means changes −0.742 ng/mL vs, −0.123 ng/mL, respectively, leading to a mean difference of −0.620 ng/mL, 95% CI −1.234 to −0.005, *p* = 0.048). Mean decrease in solCD44 at 12 months from baseline was also significantly greater with cigarettes smoked per day at baseline (0 to 5, −0.890 ng/mL, 95% CI −2.051 to 0.271, *p* = 0.037, and >10, −0.706 ng/mL, 95% CI −1.258 to −0.154, *p* = 0.049, compared to 5–10 cigarettes per day, mean change of −0.015 ng/mL, 95% CI 0.302, 0.273), and there was no significant difference comparing groups >0 to 5 versus >10 cigarettes/day (mean change −0.890 versus −0.706 ng/mL, *p* = 0.676). There was also no significant mean decrease in solCD44 at 12 months among the 21 participants who quit smoking (0 cigarettes per day at 12 months), CD44 mean change from baseline −0.049 (95%CI: −0.787 to 0.690). There was a significant reduction in cotinine levels, observed cotinine mean change −202.4 (95%CI: −396.7 to −8.1). Changes in cotinine and TP were not significantly associated with any variable by univariable analysis at *p* < 0.05 level as shown in the Supplementary Tables S2 and S3, respectively.

Multivariable models for the outcome changes in CD44, TP, and cotinine were developed testing all covariates using stepwise selection with significance level *p* < 0.30 to enter and *p* < 0.10 to stay in the models. In the CD44 multivariate model for change in solCD44 12-month vs. baseline (Table 4), only predictor at the 10% significance level were cigarettes/day at baseline (*p* = 0.062 overall, with *p* = 0.057 for regression coefficient b = 0.661 in those smoking >0 to 5 cigarettes per day versus >10 as the reference) and oral health at 12 months (*p* = 0.066 for b = −0.565 for Poor/Fair vs. Good as the reference). For cotinine and TP, the attained models confirmed the univariable analysis findings.

## 4. Discussion

An improved molecular understanding of how the cessation of smoking reduces cancer risk and how that risk can be more readily measured is needed. This work provides solutions to both. Following a smoking cessation program, participants had significantly reduced levels of tumorigenic solCD44 as measured in non-invasively acquired oral rinses.

Cotinine levels were measured to monitor exposure to nicotine in this study. Salivary levels of cotinine in individuals who do not smoke are usually less than 10 ng/mL, while levels among those who smoke a low number of cigarettes or those exposed to secondhand smoke range from 11 ng/mL to 30 ng/mL. Cotinine levels in individuals who smoke a high number of cigarettes exceed 30 ng/mL and frequently surpass 150 ng/mL but can also reach 500 ng/mL [24,25], which is consistent with our data. Here, a significant mean decrease in cotinine levels was observed, confirming that the participants were smoking fewer cigarettes at the 12-month assessment.

The main finding our study showed was a significant decrease in mean solCD44 levels following a smoking cessation program. While previous studies have compared serum or salivary CD44 levels of individuals who smoke cigarettes versus those who do not [21,26,27,28], to our knowledge this is the first occurrence of soluble CD44 being evaluated in oral rinses or saliva following participation in a smoking cessation program. While the health benefits of quitting smoking have been well researched and presented, full molecular understanding of how cellular processes respond to smoking cessation in order to restore health and reduce cancer risk is still being developed [29,30]. As shown in this manuscript, decreased levels of the tumorigenic CD44 molecule is likely one mechanism by which the human body heals.

We also investigated biomarker levels for the 21 patients who reached full cessation of cigarette smoking at the 12-month assessment. A significant decrease in mean solCD44 levels in this group was not observed, though a mean decrease in cotinine levels was significant. While a larger decrease in CD44 levels was expected, the observed outcome was likely due to 3 outlier values that negatively impacted the mean of this small 21-person cohort. While there is no justification to remove those three values, if we were to do so the mean CD44 decrease of the quitter cohort would mirror the significant decrease of the full cohort. While the mean cotinine levels of participants who quit smoking entirely decreased most when compared to all other groups (see Supplementary Table S2), our values were still elevated compared to published values. This could be explained by a lifetime of smoking and home or work secondhand smoke exposure. In addition, the amount of time that a participant had quit smoking prior to measurement of cotinine was not recorded.

Univariate analysis revealed that mean solCD44 decreased significantly more among those with poor/fair oral health than those with good oral health at baseline. Poor oral health was not associated with a higher mean change of solCD44 at 12 months when compared to baseline. This may be due to the smaller sample in the poor oral health group at 12 months compared to baseline (*N* = 45 at baseline; *N* = 41 at 12 months). Another explanation for these data concerns a subset of patients that had additional teeth removed between baseline and 12 months. Previous studies have revealed that dental treatments reduce the burden of periodontal disease, a known risk factor for cancer, and affect biomarkers of early cancer progression such as solCD44 [26,31,32]. It is possible that tooth extraction reduces periodontal disease and interferes with carcinogenesis, as our data suggests, even temporarily [33,34,35]. However, the available literature more consistently associates tooth extraction with increased cancer risk [33,36]. Case-by-case periodontal evaluation of participants before and after tooth extraction would clarify our results further.

Multivariate models assessing CD44 change at 12 months confirm association with >5 to 10 cigarettes smoked per day at baseline and poor oral health at 12 months. This data is promising and further study with larger sample sizes is warranted.

Naturally, our study has some limitations. Our results are prone to volunteer bias due to convenience sampling. Efforts were made to enroll participants at a high risk of HNC, which necessitated non-probability sampling. The dropout rate of our study was also higher than expected, possibly introducing attrition bias. Furthermore, our study design made it difficult to assess the length of time a participant had quit smoking. While 21 subjects recorded smoking 0 cigarettes at the 12-month interval, it is unknown whether they had smoked 0 cigarettes for 7 months or 7 days.

A logical next step would be to determine whether solCD44 levels are useful for monitoring dysplasia progression and resolution. Future studies could also better clarify the length of time participants had quit smoking and increase subject interval checks. Larger, randomized cohorts should also be utilized to test differences in levels of solCD44 by smoking cessation intervention type.

## 5. Conclusions

The biomarkers solCD44 and cotinine exhibited a statistically significant decrease in oral rinses after participation in a smoking cessation program in comparison to baseline, whereas TP did not. To our knowledge this is the first study to analyze these three biomarkers by salivary oral rinse after participation in a smoking cessation program. Screening solCD44 levels from salivary rinses is a promising approach to monitor decreasing HNC risk for clinicians and public health researchers.

## Figures and Tables

**Table 1:** Characteristics of subjects at baseline and at 12 months following a smoking cessation program.

	Baseline		After 12-Months		Change	Discordant Pairs, (1) → (2) (% of Change)	Discordant Pairs, (2) → (1) (% of Change)	*p*-Value (2-Tailed)
	N	%	N	%				
All	88	100.0	88	100.0	-	-	-	-
**Oral health ^c^**								
Poor/Fair (1)	45	51.1	41	46.6	−4	7 (70%)	3 (30%)	0.343 ^a^
Good (2)	43	48.9	47	53.4	4	-	-	-
**Teeth removed**								
None/1–5 (1)	43	48.9	40	45.5	−3	4 (80%)	1 (20%)	0.371 ^a^
6 or more/All (2)	45	51.1	48	54.5	3	-	-	-
**Drinking habits ^d^**								
Non-drinker/Mild (1)	16	18.2	14	15.9	−2	3 (75%)	1 (25%)	0.625 ^a^
Moderate/Heavy (2)	72	81.8	74	84.1	2	-	-	-
**Salads**								
Missing	1	1.1	1	1.1	-	-	-	-
<1/week or never (1)	35	39.8	34	38.6	−1	4 (57.1%)	3 (42.9%)	1.000 ^a^
1–3/week or more (2)	52	59.1	53	61.4	2	-	-	-
**Other vegetables**								
<1/week or never (1)	5	5.7	6	6.8	1	1 (33.3%)	2 (66.7%)	1.000 ^a^
1–3/week or more (2)	83	94.3	82	93.2	−1	-	-	-
**Green Salads or Vegetables**								
Both <1/week or never (1)	2	2.3	4	4.5	2	0 (0%)	2 (100%)	0.500 ^a^
Salads or Vegs 1–3+/week (2)	86	97.7	84	95.5	−2	-	-	-
**Green Salads & Vegetables**								
Salads/vegetables <1/week or never (1)	39	44.3	36	40.9	−3	6 (66.7%)	3 (33.3%)	0.508 ^a^
Both 1–3+/week (2)	49	55.7	52	59.1	3	-	-	-
**Cigarettes/day**								
0	0	0.0	21	23.9	+21	-	-	<0.001 ^b^
>0 to 5	17	19.3	48	54.5	+31	-	-	-
>5–10	42	47.7	14	15.9	−28	-	-	-
>10	29	33.0	5	5.7	−24	-	-	-

^a^ McNemar’s Chi-Square Test. ^b^ McNemar-Bowker Test of Symmetry. ^c^ Oral Health Score: Poor/Fair: if gingivitis or periodontitis or other oral health pathology currently present OR if last visit to dentist/dental clinic 5 or more years ago OR if last cleaning by dental hygienist was 5 or more years ago OR if teeth brushed less than once a day. Good: if no gingivitis or periodontitis or other oral health pathology currently present AND if last visit to dentist/dental clinic less than 5 years ago AND if last cleaning by dental hygienist was less than 5 years ago AND if teeth brushed more than once a day. ^d^ Drinking habits: Non-drinker/Mild: current drinking ≤2 drinks/day AND less than 2 occasions having more than 5 drinks in one occasion in the last 30 days AND past drinking of more than 2 drinks per day regularly more than 1 year ago; Moderate/Heavy: current drinking more than 2 drinks/day, OR having more than 5 drinks in one occasion in the last 30 days more than 1 time, OR past drinking regularly more than 2 drinks/day within in the last year.

**Table 2:** Salivary biomarker levels at baseline and at 12 months following a smoking cessation program.

		Baseline		At 12 Months		Change from Baseline			
	N	Mean	SD	Mean	SD	Mean	95% CI		P
CD44 (ng/mL)	88	1.816	1.280	1.405	0.963	−0.412	−0.723	−0.100	0.010
TP (mg/mL)	88	0.496	0.328	0.497	0.348	0.001	−0.085	0.088	0.975
Cotinine (ng/mL)	55	271.5	322.0	196.7	212.7	−74.7	−143.9	−5.6	0.035

P: *p*-value from paired *t* test.

**Table 3:** Selected changes in solCD44 levels at 12 months following a smoking cessation program.

Variable		CD44 Baseline		CD44 at 12 Months		Change from Baseline			
	N	Mean	SD	Mean	SD	Mean	95% CI		P
**Oral health at 12 months**									
Poor/Fair	41	2.006	1.500	1.263	0.692	−0.743	−1.222	−0.263	0.048
Good	47	1.651	1.040	1.528	1.142	−0.123	−0.528	0.283	
**Cigarettes/day at baseline**									
>0 to 5	17	2.811	1.598	1.921	1.351	−0.890	−2.051	0.271	0.048
>5–10	42	1.379	0.759	1.364	0.879	−0.015	−0.302	0.273	
>10	29	1.867	1.377	1.161	0.694	−0.706	−1.258	−0.154	
**Cigarettes/day at 12 months**									
0	21	1.896	1.185	1.847	1.355	−0.049	−0.787	0.690	0.447
>0 to 5	48	1.889	1.409	1.288	0.819	−0.601	−1.043	−0.159	
>5–10	14	1.650	1.193	1.164	0.508	−0.486	−1.165	0.193	
>10	5	1.248	0.217	1.339	0.926	0.092	−1.031	1.214	
0	21	1.896	1.185	1.847	1.355	−0.049	−0.787	0.690	0.197
>0	67	1.792	1.315	1.266	0.764	−0.525	−0.871	−0.180	

SD: standard deviation, 95% CI: 95% confidence interval, P: *p* value from two-sample t tests or from ANOVA for outcome change from baseline comparing independent groups defined by a specific variable. Note: There was no effect of age, gender, education, oral health status, teeth removed, smoking status, and drinking habits at baseline or post 12 months.

**Table 4:** Estimates regression coefficient (b) and corresponding standard error (se(b)) and *p* values (P) for univariable and multivariable models for outcome CD44 change at 12 months from baseline.

	Univariable Models			Multivariable Model *		
Variable	B	SE	P	B	SE	P
**Cigarettes/day at baseline**			0.048 ^#^			0.062 ^#^
>0 to 5	−0.184	0.439	0.676	−0.158	0.433	0.717
>5–10	0.691	0.347	0.049	0.661	0.342	0.057
>10	Reference			Reference		
**Oral health at 12 months**						
Poor/Fair	−0.620	0.309	0.048	−0.565	0.304	0.066
Good	Reference			Reference		
**Cigarettes/day at 12 months**			0.447 ^#^			
0	−0.140	0.734	0.849			
>0 to 5	−0.693	0.963	0.320			
>5–10	−0.578	0.768	0.454			
>10	Reference					
**Oral health at baseline**						
Poor/Fair	−0.583	0.309	0.063			
Good	Reference					
**Age, in years**						
<60	−0.582	0.473	0.217			
60 or more	Reference					
**Gender**						
Male	−0.194	0.316	0.540			
Female	Reference					
**Education**						
≤Grade 12 or GED	0.273	0.345	0.430			
Some college/college graduate	Reference					
**Teeth removed at baseline**						
None/1–5	−0.286	0.314	0.366			
6 or more/All	Reference					
**Teeth removed post 12 months**						
None/1–5	−0.301	0.315	0.342			
6 or more/All	Reference					
**Drinking habits baseline**						
Non-drinker/Mild	−0.199	0.370	0.592			
Moderate/Heavy	Reference					
**Drinking habits at 12 months**						
Non-drinker/Mild	0.099	0.336	0.768			
Moderate/Heavy	Reference					
**Salads at 12 months**						
<1/week or never	−0.216	0.323	0.505			
1–3/week or more	Reference					
**Green Salads & Vegs. at baseline**						
Salads/vegs <1/week or never	−0.163	0.317	0.609			
Both 1–3+/week	Reference					
**Green Salads & Vegs. post 12 months**						
Salads/vegs <1/week or never	−0.147	0.321	0.648			
Both 1–3+/week	Reference					

B: regression coefficient, SE: standard error, P: *p*-value for beta coefficient. # *p* value for effect of a variable with more than 2 categories. * Multivariable model for CD44 change, using significance level *p* = 0.30 to entry and *p* = 0.10 to stay in model, selected two variables: cigarettes/day at baseline (*p* = 0.062) and oral health at 12 months (*p* = 0.0663).

## Data Availability

Any data not presented are available upon reasonable written request.

## Supplementary Materials

The following are available online at https://www.mdpi.com/article/10.3390/ijerph182413174/s1. Table S1: Changes in CD44 levels following a 12-month smoking cessation program; Table S2: Changes in cotinine levels at 12 months following a smoking cessation program; Table S3: Changes in Total Protein levels at 12 months following a smoking cessation program.
