# Supplementary material for: Decreased Levels of Soluble CD44 in a High-Risk Population following a Smoking Cessation Program
Source: Int J Environ Res Public Health. 2021 Dec 14;18(24):13174. doi: 10.3390/ijerph182413174 (PMC8700947; doi:10.3390/ijerph182413174)
Supplement: Supplementary file 1 [file ijerph-18-13174-s001.zip › ijerph-1493665-supplementary.pdf]

Supplementary Table S1. Changes in CD44 levels following a 12-month smoking cessation program

Variable		CD44 at baseline		CD44 at 12 months		Change from baseline			
	N	*Mean*	*SD*	*Mean*	*SD*	*Mean*	*95% CI*		*P*
All patients	88	1.816	1.280	1.405	0.963	-0.412	-0.723	-0.100	0.010[#]
Age									
<60	77	1.794	1.298	1.309	0.859	-0.485	-0.825	-0.145	0.217
60 or more	11	1.974	1.190	2.077	1.377	0.103	-0.709	0.916	
Gender									
Male	47	2.034	1.517	1.532	1.091	-0.502	-1.016	0.012	0.528
Female	41	1.567	0.892	1.259	0.779	-0.308	-0.649	0.033	
Education									
<= Grade 12 or GED	62	1.634	1.037	1.303	0.836	-0.331	-0.613	-0.049	0.538
Some college/college graduate	26	2.251	1.672	1.647	1.198	-0.604	-1.459	0.251	
Oral health at baseline									
Poor/Fair	45	2.002	1.489	1.305	0.770	-0.696	-1.151	-0.242	0.063
Good	43	1.622	0.996	1.509	1.130	-0.113	-0.538	0.312	
Difference between mean changes						-0.583	-1.198	0.032	
Oral health post 12 months									
Poor/Fair	41	2.006	1.500	1.263	0.692	-0.743	-1.222	-0.263	0.048
Good	47	1.651	1.040	1.528	1.142	-0.123	-0.528	0.283	
Difference between mean of changes						-0.620	-1.234	-0.005	
Teeth removed at baseline									
None/1-5	43	2.104	1.450	1.546	1.013	-0.558	-1.115	-0.000	0.372
6 or more/All	45	1.542	1.035	1.270	0.903	-0.272	-0.588	0.044	
Teeth removed post 12 months									
None/1-5	40	2.081	1.478	1.505	1.049	-0.576	-1.171	0.019	0.366
6 or more/All	48	1.596	1.053	1.321	0.887	-0.275	-0.579	0.030	
Drinking habits at baseline									
Non-drinker/Mild	21	2.002	1.362	1.439	0.726	-0.563	-1.165	0.039	0.592
Moderate/Heavy	67	1.758	1.258	1.394	1.030	-0.364	-0.735	0.007	
Drinking habits at 12 months									

Non-drinker/Mild	29	1.678	0.872	1.332	0.763	-0.345	-0.616	-0.075	0.705
Moderate/Heavy	59	1.885	1.440	1.440	1.052	-0.444	-0.896	0.007	
Salads at 12 months									
<1/week or never	34	1.718	1.468	1.174	0.819	-0.544	-1.104	0.015	0.505
1-3/week or more	54	1.878	1.156	1.550	1.024	-0.328	-0.708	0.052	
Green Salads & Vegs. at baseline									
Salads/vegs <1/week or never	39	1.715	1.424	1.212	0.812	-0.502	-0.967	-0.038	0.609
Both 1-3+/week	49	1.897	1.161	1.558	1.051	-0.339	-0.774	0.095	
Green Salads & Vegs. post 12 months									
Salads/vegs <1/week or never	36	1.681	1.434	1.182	0.807	-0.498	-1.032	0.035	0.648
Both 1-3+/week	52	1.910	1.166	1.559	1.037	-0.351	-0.743	0.040	
Cigs/day now at baseline (q24b)									
>0 to 5	17	2.811	1.598	1.921	1.351	-0.890	-2.051	0.271	0.048
>5 -10	42	1.379	0.759	1.364	0.879	-0.015	-0.302	0.273	
>10	29	1.867	1.377	1.161	0.694	-0.706	-1.258	-0.154	
Cigs/day now at 12 months									
0	21	1.896	1.185	1.847	1.355	-0.049	-0.787	0.690	0.447
>0 to 5	48	1.889	1.409	1.288	0.819	-0.601	-1.043	-0.159	
>5 -10	14	1.650	1.193	1.164	0.508	-0.486	-1.165	0.193	
>10	5	1.248	0.217	1.339	0.926	0.092	-1.031	1.214	
0	21	1.896	1.185	1.847	1.355	-0.049	-0.787	0.690	0.197
>0	67	1.792	1.315	1.266	0.764	-0.525	-0.871	-0.180	

SD: standard deviation, 95% CI: 95% confidence interval.
P: p value from two-sample t test, except # a p value from a paired t test.

Supplementary Table S2. Changes in cotinine levels following a 12-month smoking cessation program

Variable		Cotinine at baseline		Cotinine at 12 months		Change from baseline			
	N	*Mean*	*SD*	*Mean*	*SD*	*Mean*	*95% CI*		*P*
All patients	55	271.5	322.0	196.7	212.7	-74.7	-143.9	-5.6	0.035
Age									
<60	50	264.0	301.5	183.6	174.0	-80.3	-153.4	-7.3	0.613
60 or more	5	346.4	527.6	327.6	465.3	-18.9	-344.9	307.1	
Gender									
Male	31	263.1	269.0	199.7	220.1	-63.5	-139.9	13.0	0.727
Female	24	282.2	385.9	192.9	207.5	-89.3	-220.5	41.8	
Education									
<= Grade 12 or GED	40	298.4	360.0	231.3	230.5	-67.1	-157.1	22.9	0.720
Some college/college graduate	15	199.7	176.9	104.5	118.5	-95.2	-192.7	2.3	
Oral health at baseline									
Poor/Fair	26	328.2	406.1	284.0	257.5	-44.2	-173.3	85.0	0.421
Good	29	220.6	217.0	118.5	121.1	-102.2	-171.5	-32.9	
Oral health post 12 months									
Poor/Fair	21	392.8	428.1	313.6	266.5	-79.2	-238.7	80.4	0.932
Good	34	196.5	208.4	124.5	130.1	-72.0	-135.0	-9.1	
Teeth removed at baseline									
None/1-5	25	225.6	206.9	139.2	158.3	-86.4	-176.0	3.3	0.761
6 or more/All	30	309.7	392.8	244.6	241.5	-65.0	-172.3	42.2	
Teeth removed post 12 months									
None/1-5	24	220.4	205.3	151.7	169.6	-68.7	-157.0	19.6	0.879
6 or more/All	31	311.0	388.1	231.6	237.8	-79.4	-185.8	27.0	
Drinking habits (SR Def.) baseline									
Non-drinker/Mild	11	346.2	248.5	158.5	215.9	-187.7	-312.4	-62.9	0.102
Moderate/Heavy	44	252.8	337.7	206.3	213.4	-46.5	-127.0	33.9	
Drinking habits (SR Def.) at 12 months									

Non-drinker/Mild	19	363.3	411.4	173.0	192.5	-190.3	-349.3	-31.3	0.041
Moderate/Heavy	36	223.0	256.8	209.2	224.3	-13.8	-76.0	48.5	
Difference between means						-176.5	-345.1	-8.0	
Salads at 12 months									
<1/week or never	20	310.2	406.1	195.4	189.1	-114.8	-272.5	42.9	0.452
1-3/week or more	35	249.3	266.7	197.4	227.8	-51.9	-119.7	15.9	
Green Salads & Vegs. at baseline									
Salads/vegs <1/week or never	23	287.3	383.2	213.3	199.9	-74.0	-220.3	72.4	0.986
Both 1-3+/week	32	260.1	275.9	184.8	223.9	-75.3	-140.4	-10.2	
Green Salads & Vegs. post 12 months									
Salads/vegs <1/week or never	20	310.2	406.1	195.4	189.1	-114.8	-272.5	42.9	0.452
Both 1-3+/week	35	249.3	266.7	197.4	227.8	-51.9	-119.7	15.9	
Cigs/day now at baseline (q24b)									
>0 to 5	10	90.6	93.8	83.2	98.0	-7.4	-97.1	82.2	0.550
>5 -10	26	276.6	223.2	206.9	207.2	-69.6	-166.6	27.3	
>10	19	359.6	459.4	242.5	248.6	-117.2	-272.6	38.2	
Cigs/day now at 12 months									
0	17	319.3	435.6	116.9	184.3	-202.4	-396.7	-8.1	Ref.
>0 to 5	29	192.4	191.6	206.5	183.2	14.2	-40.2	68.5	0.005
>5 -10	8	490.1	350.4	355.0	295.4	-135.0	-251.1	-18.9	0.519
>10	1	2.2	.	1.5	.	-0.7	.	.	0.422
0	17	319.3	435.6	116.9	184.3	-202.4	-396.7	-8.1	Ref.
>0 to 5	29	192.4	191.6	206.5	183.2	14.2	-40.2	68.5	0.005
>5 -10	9	435,8	350.4	365.9	315.8	-120.1	-281.0	-40.77	0.410
0	17	319.3	435.6	116.9	184.3	-202.4	-396.7	-8.1	0.067
>0	38	250.0	260.3	232.4	217.1	-17.6	-67.4	32.1	

SD: standard deviation, 95% CI: 95% confidence interval.

P: p value from two-sample t test, except # a p value from a paired t test.

Supplementary Table S3. Changes in Total Protein levels following a 12-month smoking cessation program

Variable		Total Protein baseline		Total Protein at 12 months		Change from baseline			
	N	*Mean*	*SD*	*Mean*	*SD*	*Mean*	*95% CI*		$P^{\#}$
All patients	88	0.496	0.328	0.497	0.348	0.001	-0.085	0.088	0.975
Age									
<60	77	0.490	0.331	0.473	0.294	-0.017	-0.105	0.071	0.260
60 or more	11	0.537	0.322	0.669	0.600	0.132	-0.231	0.495	
Gender									
Male	47	0.506	0.297	0.500	0.289	-0.006	-0.095	0.083	0.859
Female	41	0.484	0.364	0.494	0.410	0.010	-0.150	0.170	
Education									
<= Grade 12 or GED	62	0.462	0.273	0.486	0.350	0.025	-0.056	0.105	0.517
Some college/college graduate	26	0.577	0.428	0.523	0.350	-0.054	-0.288	0.179	
Oral health at baseline									
Poor/Fair	45	0.515	0.287	0.509	0.372	-0.007	-0.113	0.100	0.852
Good	43	0.475	0.368	0.485	0.326	0.010	-0.133	0.153	
Oral health post 12 months									
Poor/Fair	41	0.507	0.298	0.470	0.375	-0.037	-0.153	0.079	0.411
Good	47	0.486	0.355	0.521	0.326	0.035	-0.095	0.165	
Teeth removed at baseline									
None/1-5	43	0.584	0.353	0.589	0.295	0.005	-0.137	0.148	0.929
6 or more/All	45	0.412	0.281	0.409	0.375	-0.002	-0.109	0.104	
Teeth removed post 12 months									
None/1-5	40	0.588	0.360	0.573	0.303	-0.015	-0.168	0.137	0.734
6 or more/All	48	0.419	0.280	0.434	0.373	0.015	-0.085	0.116	
Drinking habits at baseline									
Non-drinker/Mild	21	0.507	0.289	0.409	0.237	-0.099	-0.210	0.013	0.090
Moderate/Heavy	67	0.492	0.341	0.525	0.374	0.033	-0.076	0.141	
Drinking habits at 12 months									
Non-drinker/Mild	29	0.472	0.282	0.416	0.247	-0.056	-0.152	0.041	0.271
Moderate/Heavy	59	0.507	0.350	0.537	0.384	0.029	-0.092	0.151	
Salads at 12 months									
<1/week or never	34	0.483	0.419	0.419	0.299	-0.064	-0.223	0.095	0.237

1-3/week or more	54	0.504	0.259	0.546	0.370	0.043	-0.060	0.145	
Green Salads & Vegs. at baseline									
Salads/vegs <1/week or never	39	0.483	0.303	0.503	0.439	0.021	-0.112	0.153	0.697
Both 1-3+/week	49	0.506	0.350	0.492	0.260	-0.014	-0.133	0.105	
Green Salads & Vegs. post 12 months									
Salads/vegs <1/week or never	36	0.496	0.410	0.409	0.294	-0.087	-0.240	0.066	0.092
Both 1-3+/week	52	0.496	0.261	0.558	0.372	0.063	-0.040	0.165	
Cigs/day at baseline									
>0 to 5	17	0.695	0.510	0.637	0.360	-0.058	-0.412	0.295	0.802
>5 -10	42	0.461	0.264	0.476	0.387	0.015	-0.097	0.127	
>10	29	0.429	0.231	0.446	0.261	0.017	-0.075	0.108	
Cigs/day at 12 months									
0	21	0.480	0.312	0.594	0.501	0.113	-0.107	0.334	0.495
>0 to 5	48	0.550	0.364	0.498	0.293	-0.052	-0.175	0.072	
>5 -10	14	0.367	0.225	0.337	0.177	-0.029	-0.137	0.079	
>10	5	0.401	0.169	0.527	0.365	0.126	-0.287	0.539	
0	21	0.480	0.312	0.594	0.501	0.113	-0.107	0.334	0.151
>0	67	0.501	0.335	0.467	0.283	-0.034	-0.126	0.059	

SD: standard deviation, 95% CI: 95% confidence interval.

P: p value from two-sample t tests, except # a p value from a paired t test.
